# Effects of selected bioactive food compounds on human white adipocyte function

**DOI:** 10.1186/s12986-016-0064-3

**Published:** 2016-01-19

**Authors:** Christel Björk, Uta Wilhelm, Susanne Mandrup, Bjørk Ditlev Larsen, Alessandra Bordoni, Per Hedén, Mikael Rydén, Peter Arner, Jurga Laurencikiene

**Affiliations:** Lipid Laboratory, Department of Medicine, Karolinska Institutet, Huddinge, Stockholm, Sweden; Department of Biochemistry and Molecular Biology, University of Southern Denmark, 5230 Odense M, Denmark; Department of Agro-Food Sciences and Technologies, University of Bologna, Bologna, Italy; Department of Plastic Surgery, Akademikliniken, Stockholm, Sweden; Department of Medicine, Karolinska Institutet, Lipid Laboratory, Novum, NVS D4, Hälsovägen 7, 14186 Stockholm, Sweden

**Keywords:** Anthocyanin, Beta-glucan, Cyanidin-3-glucoside, Docosahexaenoic acid, Inflammation, Insulin sensitivity, Lipolysis, Propionic acid, Protocatechuic acid

## Abstract

**Background:**

Previous studies suggest that intake of specific bioactive compounds may have beneficial clinical effects on adipose tissue partly due to their anti-inflammatory and insulin-sensitizing properties. With the overall aim to contribute to better understanding of the mechanisms of selected bioactive nutrients on fat metabolism, we investigated their role on human white adipocyte function.

**Methods:**

The influence of the omega-3-fatty acid docosahexaenoic acid (DHA)**,** the anthocyanin (AC) cyanidin-3-glucoside and its metabolite protocatechuic acid, and the beta-glucan metabolite propionic acid (PI) on adipokine secretion, fatty acid metabolism (lipolysis/lipogenesis) and adipocyte differentiation (lipid accumulation) was studied in human fat cells differentiated in vitro. To investigate possible synergistic, additive or antagonistic effects, DHA was also combined with AC or PI.

**Results:**

Each compound, alone or together with DHA, suppressed basal adipocyte lipolysis compared to control treated cells. DHA alone attenuated the secretion of pro-inflammatory adipokines such as chemerin, interleukin-6 (IL-6) and monocyte chemoattractant protein-1 (MCP-1/*CCL2*), whereas AC suppressed only the latter two. Treatment with PI decreased IL-6, tumour necrosis factor alpha (TNFα) and adiponectin secretion. A combination of DHA and AC decreased TNFα secretion and increased insulin-stimulated lipogenesis. No effect was found on adipocyte differentiation. At the selected concentrations, none of the compounds was found to be cytotoxic.

**Conclusion:**

The studied bioactive food compounds or their metabolites have beneficial effects in human primary fat cells measured as decreased basal lipolytic activity and secretion of inflammatory markers. A minor effect was also observed on insulin-stimulated glucose uptake albeit only with the combination of DHA and AC. Taken together, our results may link the reported health benefits of the selected bioactives on metabolic disorders such as insulin resistance, hypertension and dyslipidemia to effects on white adipocytes.

**Electronic supplementary material:**

The online version of this article (doi:10.1186/s12986-016-0064-3) contains supplementary material, which is available to authorized users.

## Background

It is well established that white adipose tissue (WAT) metabolism and inflammatory status affect whole body homeostasis [1]. The major role of WAT is to store and release energy. Storing energy in the form of triacylglycerol (TAG) in adipocytes through lipogenesis has been shown to regulate systemic insulin sensitivity [2, 3]. Hydrolysis of TAGs into non-esterified fatty acids and glycerol is a tightly controlled enzymatic process denoted lipolysis. Basal (spontaneous) lipolytic activity is increased in obesity and may attenuate insulin sensitivity through increased delivery of FAs to skeletal muscle (reviewed in [4]). Another important aspect of WAT function is the secretion of a class of proteins called adipokines, several with pro- or anti-inflammatory properties (see [1] for review). An elevated release of monocyte chemoattractant protein-1 (MCP-1/ *CCL2*) produced by adipocytes [5, 6] recruits macrophages, which in turn promote a chronic low grade inflammation in WAT [7]. Release of other pro-inflammatory adipokines, such as interleukin-6 (IL-6), tumour necrosis factor-alpha (TNFα) and chemerin may also contribute to metabolic disorders such as insulin resistance, dyslipidemia, hypertension and cardiovascular disease [8–10], which are associated with the development of the metabolic syndrome [11]. The anti-inflammatory adipokine adiponectin has on the other hand been shown to correlate negatively with BMI (Body mass index, defined as body weight in kilograms divided by height in meters squared) and positively with insulin sensitivity [12, 13] and its high levels are associated with a lower risk for developing type 2 diabetes [14].

Diet composition can affect the metabolic and endocrine function of WAT as well as overall energy balance [15]. Certain diets that provide high amounts of bioactive components may have beneficial clinical effects partly due to their anti-inflammatory effects on adipose tissue (see [15, 16] for reviews). A number of natural bioactive compounds that can be added to the food or their metabolic derivatives have been tested for their action on fat cells. The omega-3 long-chain polyunsaturated fatty acid docosahexaenoic acid (DHA) can be synthesized from the essential alpha-linoleic acid. However, minor (clinically insufficient) amounts are synthesized endogenously and significant levels can only be provided exogenously through intake of fish and fish oil. A previous study using human adipocytes and WAT suggested an anti-inflammatory effect of DHA via decreased levels of IL-6 and MCP-1 [17]. In vitro studies regarding the regulation of DHA on adiponectin production have been performed both in mouse and human adipocytes, however the results are conflicting [18–20]. Anthocyanins (AC) are naturally occurring polyphenol compounds present in blue or purple berries, fruit and vegetables [21]. Cyanidin-3-glucoside (C3G) is the most studied anthocyanin that together with its main metabolite protocatechuic acid (PCA) has been shown to be protective against insulin resistance in both human adipocytes in vitro [22, 23] and an in vivo diabetic mouse model [24, 25] as well as having anti-inflammatory properties [22, 24–26]. Beta-glucan (BG) is a soluble dietary fiber present in oats and barley. However, BG is not absorbed in vivo*,* but its metabolism by the gut microbiota results in increased production an uptake of short-chain fatty acids (SCFA), such as propionic acid (PI), which has been shown to regulate adipokine production and enhance lipogenesis and glucose uptake in isolated human WAT [27, 28].

Most studies investigating effects of these compounds have been conducted in murine adipose cell lines or in vivo mouse or rat models [29–32], which display a number of species-specific differences concerning adipose tissue function [33–35]. Furthermore, in previous studies the compounds have been tested individually and the experimental conditions as well as fat cell model systems have varied markedly. In addition, in most cases a single metabolic or inflammatory aspect of adipocyte function has been investigated.

As a part of the pan-European study PATHWAY-27 (http://www.pathway27.eu/) with the overall aim to contribute to better understanding of the mechanisms of bioactive nutrients on metabolism in different food-environments and help to develop health-promoting food for the growing demand of the health-conscious consumer, we have investigated the role of selected bioactives or their main metabolites on adipocyte phenotypes linked to obesity and the metabolic syndrome.

## Methods

### Cell cultures of primary human adipocytes

Primary human pre-adipocytes for in vitro differentiation from 22 female subjects and 2 male subjects between 26-65 (mean 42) years of age with a BMI ranging from 21-31 kg/m^2^ (mean 25) were isolated from the stromal vascular fraction of subcutaneous WAT, which was obtained as a waste product from cosmetic liposuction. Isolation, culture and differentiation into adipocytes were performed as previously described [36]. The subjects were healthy according to self-report and not selected for age, sex or BMI. Briefly, intact pieces of adipose tissue were cut into 3-4 mm pieces and digested with collagenase for 90 min at 37 °C, whereas material obtained from liposuctions was digested with collagenase for 45 min at 37 °C. Thereafter the cell suspension was filtered, centrifuged at 200 × g for 10 min. The remaining pre-adipocytes and mesenchymal stem cells in the stromal vascular fraction were then suspended in erythrocyte lysis buffer for 10 min and centrifuged. The supernatant was discarded, the cells suspended in proliferation medium containing Dulbecco’s Modified Eagle Medium (DMEM)/F12 (Biochrome AG, Berlin, Germany/Gibco, Life Technologies) supplemented with 10 % fetal bovine serum (Gibco, Life Technologies) and 2 % Pen-Strep (Gibco, Life Technologies) and subsequently filtered through a 70 μm cell strainer (BD BioSciences, Durham, NC) .

To study the effects of bioactives on fat cell metabolism and adipokine secretion, the cells were plated in proliferation medium at a density of 115 000 to 120 000 cells/well in 24-well plates. After 24 h, the medium was changed to DMEM/F12 supplemented with 15 mM HEPES (Gibco, Life Technologies), 100 μg/ml penicillin-streptomycin (Gibco, Life Technologies), 66 nM human insulin, 1 nM triiodo-L-thyronine, 10 μg/ml human transferrin, 33 μM biotin, 17 μM panthothenate, 100 nM cortisol (all from Sigma St. Louis, MO), 1.25 μg/ml amphotericin B (Gibco, Life Technologies) and 10 μM rosiglitazone (BRL49653) (GlaxoSmithKline, Durham, UK) to induce differentiation. Rosiglitazone was included during the first 3-4 days and then removed from the differentiation medium. Medium was changed every 2-3 days and the cells were kept until full differentiation (12-14 days). Forty-eight hours before full differentiation, the compounds were added in adipogenic medium.

To investigate the effects of bioactives on lipid accumulation, 20 000 cells/well were seeded in 96-well plates. Medium was supplemented with the compounds after 6 days in differentiation medium and treatment was performed for 6 days until day 12 of differentiation. Adipose tissue stromal vascular fraction, which we use to in vitro differentiate adipocytes, contains various cell types including immune cells. At day 6 of differentiation, immune cells are almost entirely washed away and absent and this is the earliest time point in differentiation, where effects unspecific for adipocytes could be excluded. Each experiment was repeated in cells isolated separately from at least three individuals in duplicates (lipid accumulation and lipogenesis) or quadruplicates (lipolysis and adipokine secretion). Cytotoxicity of compounds was measured for all treatment conditions. The grade of differentiation was determined under the microscope and cells with differentiation grade below 80 % were discarded. The study was approved by the Ethics Committee at Karolinska Institutet (Sweden). All subjects were informed about this study and provided their written informed consent.

### Treatment with bioactive compounds

The cells were treated with DHA (D8768, sodium salt, Sigma, St. Louis, MO)**,** C3G (1201-1, chloride salt, Polyphenols Laboratories AS, Sandes, Norway) together with its metabolite PCA (03930590, Sigma) or the BG metabolite PI (P1880, sodium salt, Sigma). To study possible synergistic, additive or antagonistic effects with DHA, the bioactive compound was also studied in combination with AC or PI. Compound concentrations were based on relevant literature [27, 37] and unpublished treatment optimizations in the human Simpson-Golabi-Behmel syndrome (SGBS) pre-adipocyte cell line or in a hepatocyte cell line by collaborators in the PATHWAY-27 consortium. DHA was coupled to bovine serum albumin (BSA; A6003, Fraction V, Sigma) in a 4:1 ratio. Firstly, BSA was dissolved in 150 mM NaCl in a 37 °C water bath and filtered via a sterile 0.2 μm polypropylene membrane (VWR International, USA). Shortly after, DHA was dissolved in water and heated to 70 °C in a water bath. The coupling was performed through mixing both heated solutions and stirring for 1 h at 37 °C (pH 7.4). The final DHA concentration in the cell culture medium was 0.5-60 μM. C3G was dissolved in phosphate-buffered saline (PBS, Gibco, Life Technologies) whereas its main metabolite PCA was diluted in 50 % v/v solution of ethanol in water. According to agreements within the PATHWAY-27 consortium, C3G and PCA were added together to represent AC in a final concentration of 130 nM and 13 μM, respectively. PI (P1880, sodium salt, Sigma) was dissolved in 50 % v/v solution of ethanol in water and added to the cells at a final concentration of 100 μM. Corresponding concentrations of BSA and EtOH were added the control samples. As higher concentrations of BSA has been reported to have stimulatory effect on inflammatory cytokine secretion [38], we have performed control experiments comparing BSA and EtOH (at concentrations used in all our treatments) to untreated cells. Such treatment did not affect release of IL-6 and glycerol (Additional file 1: Figure S1).

### Cytotoxicity measured as lactate dehydrogenase (LDH) activity in conditioned medium

Medium from bioactive treated cells was collected when full differentiation was reached. LDH activity, as a measure of damaged cells, was quantified according to the manufacturer’s instructions (Cat. No. 11 644 793 001, Roche Diagnostics GmbH, Mannheim, Germany).

### Lipolysis assay

Conditioned medium was collected after 48 h of treatment with the bioactive compounds. Basal lipolytic activity was measured as glycerol release with a bioluminescence method using Free Glycerol determination kit from Sigma (FG100) in combination with the fluorescence probe Amplex ultra red reagent (Cat. No. A36006, Molecular Probes, Life Technologies) [39, 40]. Glycerol release was normalized to protein concentration in each cell culture well.

### Lipogenesis

Determination of basal and insulin-stimulated glucose incorporation into lipids was made when cells reached full differentiation that is after 48 h of bioactive treatment. Cells were initially washed twice with insulin- and glucose free DMEM (BioChromeAG) and incubated in DMEM containing 1 μM glucose. After 3 h, 3-^3^H glucose (37 MBq/ml; Perkin Elmer-Cetus, Norwalk, CT) diluted 1:1000 in DMEM containing 1 μM glucose with was added. For stimulated lipogenesis, insulin was added to a final concentration of 100 nM. After 2 h of incubation, the cells were washed three times with 4 °C PBS and thereafter lysed in aqueous 0.1 % SDS (sodium dodecyl sulfate, Sigma) solution. An aliquot was saved for protein determination and the rest of the lysate was transferred to scintillation tubes. Toluene scintillation liquid (toluene with 5 g/l 2,5-diphenyloxazol and 0.3 g/l 1,4-bis (4-methyl-5-phemyl-2-oxazolyl)-benzene; Sigma-Aldrich) was added and the samples were incubated overnight to extract the lipids. Glucose incorporation into lipids as a proxy of lipogenesis was quantified as 3-^3^H counts per minute (CPM) using a LS 6500 Multi-Purpose scintillation counter (Beckman, USA).

### Adipokine quantification by enzyme-linked immunosorbent assay (ELISA)

To quantify the secretion of pro- (IL-6, MCP-1, chemerin, and TNFα) and anti-inflammatory (adiponectin) adipokines, cells were treated with bioactive compounds for 48 h and conditioned medium was collected for adipokine quantification by ELISA according to the manufacturer’s instructions. The samples were diluted 5-60 fold for IL-6 detection (Quantikine ELISA Human IL-6 Immunoassay; R&D Systems, Minneapolis, MN, Intra-assay precision: CV <5 %, Inter-assay precision: CV <4 %), 3-10 fold for MCP-1 (Quantikine ELISA Human CCL3/MCP-1 Immunoassay; R&D Systems, Intra- and Inter-assay precision: CV <6 %) and 2-10 fold for chemerin (Quantikine ELISA Human Chemerin Immunoassay; R&D Systems, Intra-assay precision: CV ≤3 %, Inter-assay precision: CV <6 %) to match the standard curve. Adiponectin (Mercodia Adiponectin ELISA; Mercodia AB, Uppsala, Sweden, Intra-assay precision: CV <5 %, Inter-assay precision: CV <8 %) and TNFα (Quantiglo ELISA Human TNFα Immunoassay; R&D Systems, Intra-assay precision: CV <6 %, Inter-assay precision: CV <9 %) secretion were measured in undiluted samples. Levels of TNFα were detectable in adipocyte cultures treated with AC from only two subjects.

### Protein quantification

Protein amount was quantified in SDS lysates from the lipogenesis experiments using Pierce™ BCA Protein Assay Kit (Cat. No. 23225 Pierce, Rockford, IL) according to the manufacturer’s instructions. The protein concentration for each well was used to normalize glucose incorporation into lipids as well as glycerol release, LDH activity and adipokine secretion for cell amount.

### Neutral lipid and DNA staining

The cells were differentiated in 96-well plates and treated with the bioactive compounds for 6 days before full differentiation. Medium supplemented with the compounds was added to the cell cultures every second day. Before staining, the cells were washed with PBS and fixed with 4 % paraformaldehyde solution (PFA) containing 0.123 M NaH_2_PO_4_xH_2_O, 0.1 M NaOH and 0.03 M glucose for 10 min at room temperature. Fixed cells were washed with PBS and stained with Hoechst 33342 (2 μg/ml; staining cell nuclei, Cat. No. H3570, Molecular probes) and Bodipy 493/503 (0.2 μg/ml; staining neutral lipids, Cat. No. D-3922, Molecular probes) diluted in PBS for 20 min at room temperature. After washing with PBS, accumulation of intracellular lipid droplets and cell number (stained nuclei) were quantified with Acumen eX3 imager (TTP Labtech, Hertfordshire, UK). Bodipy lipid droplet fluorescence was normalized to the amount of nuclei in each well (Hoechst).

### Statistical analysis

The impact of DHA, AC and PI on cytotoxicity, lipid accumulation, adipokine secretion and metabolic outcomes (lipolysis/lipogenesis) was compared to control cells without bioactives. If statistical significance was reached, the effect was also compared with the other bioactives having significant outcomes. Additionally, to investigate possible combinatorial (synergistic, additive or antagonistic) effects of DHA exposure, the impact of DHA alone was compared to that obtained in combination with AC or PI. If a difference was found, AC and PI exposure alone was compared to the effect of the respective bioactive in combination with DHA. The data was tested for normal distribution with the Shapiro-Wilk test. If the criteria for normal distribution were fulfilled, one-way analysis of variance (ANOVA) with subsequent Tukey’s HSD post-hoc test was utilized. When the criteria for normal distribution were not achieved, the non-parametric Kruskal-Wallis and Mann-Whitney pair-wise comparison tests were used. The level of statistical significance was set as 0.05 with *p < 0.05, **p < 0.01 and ***p < 0.001. Bars are shown as mean ± standard deviation.

## Results

### Titration of the in vitro conditions for DHA treatment of primary human adipocytes

DHA concentrations that have been used in other fat cell systems (10-60 μM) [18–20, 41] as well as in optimizations by PATHWAY-27 collaborators in SGBS cells, induced LDH activity in conditioned media (after 6 days of stimulation) and increased IL-6 secretion (after 48 h of stimulation), indicating both cytotoxic and pro-inflammatory effects (Additional file 2: Figure S2). These findings are difficult to reconcile with the reported physiological effects of omega-3-fatty acids in vivo [42]. Consequently, the DHA concentration was titrated down and 0.5 μM was selected as the highest non-toxic DHA concentration in our primary culture system.

### Decreased basal adipocyte lipolysis after treatment with bioactive compounds

**Fig. 1:**
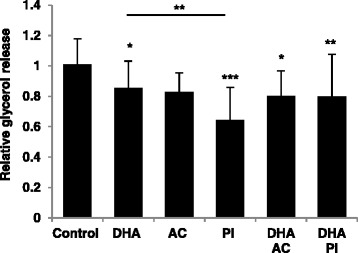
Decreased basal adipocyte lipolysis after treatment with bioactive compounds or metabolites. Glycerol release after 48 h of treatment with 0.5 μM docosahexaenoic acid (DHA), anthocyanin (AC), that is 130 nM cyanidin-3-glucoside (C3G) and 13 μM protocatechuic acid (PCA), and 100 μM propionate (PI), relative to control, measured in conditioned medium from human in vitro differentiated adipocytes. Control, n = 9; 0.5 μM DHA, n = 6; AC alone and with DHA, n = 3; 100 μM PI alone and with DHA, n = 4 biological/independent experiments in quadruplicates. Normalized data is adjusted for protein amount and presented as means +/- standard deviation. *p < 0.05, **p < 0.01 and ***p < 0.001 versus control. Statistical significance was determined by one-way ANOVA with Tukey’s multiple comparisons post hoc test

Glycerol release was significantly decreased after all treatments when compared to control, with the exception of AC (p = 0.056) (Fig. 1). Treatment with PI had the strongest attenuating effect on glycerol release compared to control-treated cells which was stronger than that of DHA (p = 0.009). None of the compounds induced cytotoxicity at the selected concentrations, but rather reduced LDH activity in cell cultures after 48 h of treatment (Additional file 3: Figure S3), except for AC in combination with DHA (p = 0.321, due to a large inter-individual variation of the response). LDH activity remained unaltered up after 6 days of treatment with the bioactive compounds.

### Combined treatment with DHA and AC increased insulin-stimulated adipocyte lipogenesis

**Fig. 2:**
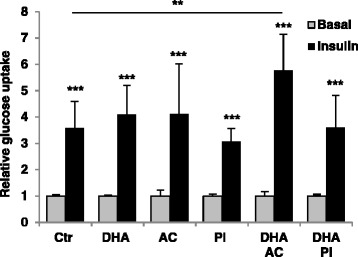
Combined treatment with DHA and AC increased insulin-stimulated adipocyte lipogenesis. Basal and insulin-stimulated lipogenesis measured in cell lysates after 48 h of treatment with bioactive compounds. Control, n = 8; 0.5 μM DHA, n = 6; AC alone and with DHA, n = 3; 100 μM PI alone, n = 3 and with DHA, n = 4 biological/independent experiments in duplicates. Glucose incorporation into lipids (3-^3^H counts per minute) is normalized for protein concentration in the cell lysates for each well. Normalized data is presented as means +/- standard deviation. *p < 0.05, **p < 0.01 and ***p < 0.001 counted against basal control with the same bioactive treatment or insulin-stimulated control compared with insulin-stimulated bioactive treated cells. Statistical significance was determined by the non-parametric Kruskal-Wallis test with subsequent Mann-Whitney multiple comparisons post hoc test

To determine the effects of the bioactive compounds or their metabolites on insulin-stimulated lipogenesis, relative glucose incorporation into lipids was measured in the in vitro differentiated primary adipocytes. Insulin induced lipogenesis in the control and all treatment conditions (Fig. 2). No effect on insulin-stimulated response was found after treatment with any of the compounds alone.

However, combined treatment with DHA and AC increased insulin-stimulated glucose uptake compared to the control.

### Bioactives alter secretion of pro-inflammatory adipokines and adiponectin

**Fig. 3:**
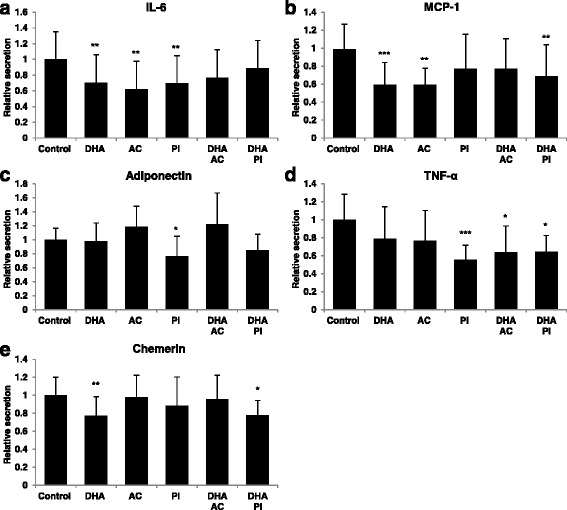
Bioactives or metabolites alter secretion of pro-inflammatory adipokines and adiponectin. Secretion of adipokines; interleukin-6 (IL-6) (**a**), monocyte chemoattractant protein-1 (MCP-1) (**b**), adiponectin (**c**), tumour necrosis factor α (TNFα) (**d**) and chemerin (**e**); after 48 h of treatment with bioactive compounds relative to control in conditioned media. Control, n = 6-9; 0.5 μM DHA, n = 3-6; AC alone and with DHA, n = 3; 100 μM PI alone and with DHA, n = 3-4 in biological/independent experiments in quadruplicates, with the exception of TNFα where n = 2-6 due to levels below minimum detectable dose in four subjects. Normalized data is adjusted for protein amount and presented as means +/- standard deviation. *p < 0.05, **p < 0.01 and ***p < 0.001 versus control. Statistical significance was obtained by one-way ANOVA with Tukey’s multiple comparisons post hoc test

To investigate the effect of the bioactive compounds or their metabolites on a panel of the most important pro- (IL-6, MCP-1, TNFα, chemerin) and anti-inflammatory (adiponectin) adipokines, conditioned media from the adipose cells were collected after 48 h of treatment and secretion was measured by ELISA. IL-6, MCP1 and chemerin secretion were suppressed by DHA (Fig. 3a, b and e). The effect of DHA on MCP-1 and chemerin secretion was also statistically significant in combination with PI (Fig. 3b and e). Additionally, DHA combined with PI impeded TNFα secretion, a result that was also obtained with PI alone (Fig. 3d). PI was the only compound that had an effect on adiponectin, contributing to a slightly decreased secretion (Fig. 3c). As the other two compounds, PI suppressed IL-6 secretion (Fig. 3a). AC alone attenuated IL-6 and MCP-1 secretion and in combination with DHA, demonstrated a suppressed secretion of TNFα (Fig. 3a, b and d).

### No effects of bioactives on adipocyte differentiation

**Fig. 4:**
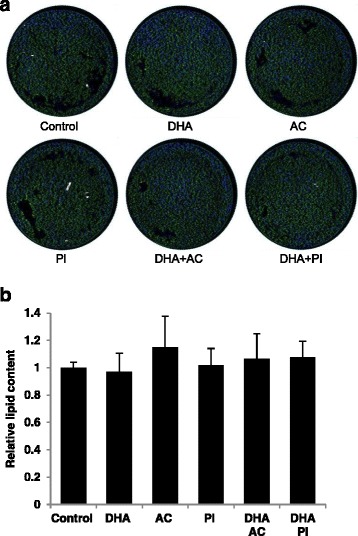
No effects of bioactives or metabolites on lipid accumulation. Representative pictures of neutral lipid accumulation (green) and cell nuclei (blue) staining after 6 days of treatment with bioactive components (**a**). Quantified lipid accumulation adjusted for cell amount (control, n = 8; 0.5 μM DHA, n = 8; AC alone and with DHA, n = 4; 100 μM PI alone and with DHA, n = 6 biological/independent experiment in duplicates (**b**). Normalized data is presented as means +/- standard deviation versus control. The absence of statistical significance was determined by the non-parametric Kruskal-Wallis test

To measure possible effect of bioactives on adipocyte differentiation and lipid accumulation, cells were treated for 6 days and lipid content was measured by Bodipy staining and normalized per cell based on DNA staining by Hoechst (Fig. 4a). No effects were found of the selected compounds on lipid accumulation after 6 days of treatment (Fig. 4b).

## Discussion

Using a systematic assessment of the effects of specific bioactive food compounds and their metabolites in human white fat cells, we found beneficial effects of DHA, AC and PI, either alone or in combination with DHA, on fat cell metabolism and inflammatory phenotype in vitro. The in vitro differentiated non-expanded human primary adipocytes used herein, is the closest model to human fat cells in vivo and enables longer treatments with the compounds. Treatments in in vitro systems always raise a question on the physiological concentrations of the stimuli applied and relevance of the resulting effects for the in vivo situation. We utilized lower concentrations of DHA than that measured in blood [43, 44]. However, higher concentrations of DHA induced cytotoxicity and pro-inflammatory response as in previous studies [20, 45]. Since the formation of reactive oxygen species (ROS) correlates with the number of double bonds in a polyunsaturated fatty acid, DHA is highly susceptible to spontaneous oxidation. ROS activate surface receptors such as Toll-like receptors which can trigger a downstream signalling cascade, leading to activation of the NF-kappaB pathway, which then turn on the transcription of many pro-inflammatory genes [46, 47]. In the support of this hypothesis, ascorbic acid suppressed the pro-inflammatory effect in our primary human adipocyte culture (data not shown). Furthermore, total plasma levels of DHA should not be interpreted as equivalent to local extracellular or intracellular concentrations. To the best of our knowledge, there are no studies that have measured the actual concentration of DHA in adipose tissue. However, we cannot exclude that in the in vivo environment higher concentrations of DHA would be needed for optimal effects. Additionally, none of the other compounds displayed cytotoxicity in our cell cultures and the concentrations used for PI and AC were close to those reported in human blood [48, 49].

All three compounds suppressed basal adipocyte lipolysis, where the effect of PI was slightly more pronounced compared with DHA and AC. This could at least in part be due to a fact that PI also significantly attenuated the secretion of TNFα, a potent inducer of adipocyte lipolysis [33]. Our finding corroborate results from a study where healthy subjects were supplemented for 4 weeks with non-digestible starch and demonstrated decreased adipose tissue glycerol release as well as reduction in the activity of hormone-sensitive lipase, the rate-limiting enzyme responsible for mobilization of stored TAGs [49]. We therefore suggest that PI might exert its effects at least in part by altering adipocyte lipid metabolism via indirect (through TNFα) or direct mechanisms. In fact, although the latter mechanism has not been not studied in humans, in mice PI has been suggested to interact with the G-protein coupled receptor 43 [29], a receptor that is suggested to be involved in regulating inflammatory responses, and is also expressed in human WAT [28]. Regarding ACs, an anti-lipolytic effect has been observed in 3T3-L1 adipocytes during hyperglycemia, where C3G regulated FoxO1-mediated transcription of adipose triglyceride lipase resulting in decreased expression of this lipolytic enzyme [50]. Whether or not this also implies for human adipocytes remains to be investigated. The mechanism of lipolysis reduction by DHA is not known. However, no additive effects between compounds were observed, suggesting that they might act via similar mechanisms.

The synergistic effect between DHA and AC on insulin-induced lipogenesis might partly be explained by the activation of PPARγ by AC, which results in increased expression and translocation of GLUT4 [22]. In addition, the effect on lipogenesis could result from decreased TNFα secretion caused by combined DHA and AC treatment. We and others have shown that TNFα addition in cultures of primary human adipocytes causes insulin resistance [51, 52]. However, a combination of PI and DHA also lowered TNFα secretion but did not affect lipogenesis, which may suggest different mechanisms of action.

Earlier studies both in mice WAT and human adipocytes have shown suppression of pro-inflammatory cytokines by AC [24–26], DHA [17] and PI [27], effects that are generally corroborated in the current study. DHA appears to be the strongest anti-inflammatory compound among the tested ones as this fatty acid decreased expression of three out of four investigated pro-inflammatory cytokines. We also for the first time report effects of bioactive compounds on chemerin – an insulin resistance inducing cytokine [8], which is largely secreted by adipocytes. DHA was the only compound suppressing chemerin secretion, which was antagonized by AC. Admittedly, at present we do not know how the selected bioactives regulate adipokine secretion, a question which needs further investigations. The outcome of compound interaction could also depend on their concentrations, the effects might not be linear and additional experiments using a concentration-dependent response for each bioactive alone and in combination are warranted to better establish any possible additive/synergistic effects on human adipocytes. However, our data suggest interactions between bioactives on fat cell function, which is relevant for in vivo physiology and nutrition.

Publications from murine 3T3-F442A and -L1 fat cells as well as isolated primary human adipocytes have demonstrated increased adiponectin secretion after DHA treatment [18, 19, 53]. However, it should be noted that fetal bovine serum, an additive which may protect the cells from ROS exposure, was included in the cell culture medium in all these previous studies. This may explain why higher concentrations of DHA could be used in those experiments. Other possible reasons for the discrepancies may be treatment duration or delivery as in vitro cell systems seem to be sensitive to this compound. The effect of PI on adiponectin secretion was unexpected, but could be due to its effect on PPARγ reported previously [54]. On the other hand we did not observe any effects of the compounds on fat cell differentiation/lipid accumulation, which speaks against their possible role regulating important adipogenic factors at least in in vitro cell system.

## Conclusions

Our systematic assessment of DHA, AC and PI, either alone or in combination with DHA, indicate a beneficial role on human fat cell function. The selected compounds decreased basal lipolytic activity and secretion of inflammatory markers in human fat cells which might explain some of the reported health benefits induced by bioactive compound-containing diets in humans, including reduced insulin resistance, hypertension and dyslipidemia [21, 42, 55]. To further elucidate this question, our in vitro results will be used to identify specific biomarkers to be measured in volunteers recruited in a clinical intervention study within the PATHWAY-27 project.

## Additional files

Additional file 1: Figure S1. No effect of BSA and EtOH on glycerol release or IL-6 secretion. Glycerol release (a) and IL-6 secretion (b) after 48 h of treatment with 0.05 % Ethanol (EtOH) and 0.125 μM bovine serum albumin (BSA) relative to untreated cells in conditioned media (n = 2 biological/independent experiments in quintuplicates). Normalized data is adjusted for protein amount and presented as means +/- standard deviation. The absence of statistical significance was determined by Tukey’s test. (PPTX 46 kb) Additional file 2: Figure S2. Adverse effects of DHA on adipocyte cytotoxicity and IL-6 secretion. Cytotoxicity detection measured as lactate dehydrogenase (LDH) activity in conditioned media after 6 days of treatment with 0.5 μM, 10 μM or 60 μM DHA, relative to control (a). Control, n = 7; 0.5 μM DHA, n = 3; 10 μM and 60 μM DHA, n = 2 biological/independent experiments in at least duplicates. Secretion of IL-6 from human in vitro differentiated adipocytes after 48 h of treatment with 0.5 μM, 5 μM, 10 μM, 20 μM, 30 μM or 60 μM DHA compared to control (b). Control, n = 2 and DHA n = 1 biological/independent experiments in triplicates. Normalized data is adjusted for protein amount and presented as means +/- standard deviation. **p < 0.05*, ***p < 0.01* and ****p < 0.001* versus control. Statistical significance was obtained by one-way ANOVA with Tukey’s multiple comparisons post hoc test. (PPTX 47 kb) Additional file 3: Figure S3. Decreased cytotoxicity after treatment with bioactive compounds or metabolites. Lactate dehydrogenase (LDH) activity after 48 h or 6 days treatment with bioactive compounds relative to control in conditioned media. Forty-eight hours; control, n = 9; 0.5 μM DHA, n = 6; AC alone and with DHA, n = 3; 100 μM PI alone and with DHA, n = 4 biological/independent experiments in quadruplicates; 6 days; control, n = 7; 0.5 μM DHA, n = 7; AC alone and with DHA, n = 4; 100 μM PI alone and with DHA, n = 5 biological/independent experiments in duplicates. Normalized data is adjusted for protein amount in each well as a measure of cell amount and presented as means +/- standard deviation. **p < 0.05*, ***p < 0.01* and ****p < 0.001* versus control. Statistical significance was determined by one-way ANOVA with Tukey’s multiple comparisons post hoc test. (PPTX 44 kb)

## Abbreviations

AC: anthocyanin
BG: beta-glucan
BMI: body mass index
BSA: bovine serum albumin
C3G: cyanidin-3-glucoside
CPM: counts per minute
DHA: docosahexaenoic acid
ELISA: enzyme-linked immunosorbent assay
LDH: lactate dehydrogenase
IL-6: interleukin-6
MCP-1/ *CCL2*: monocyte chemoattractant protein-1
PCA: protocatechuic acid
PI: propionic acid
PFA: paraformaldehyde solution
PBS: phosphate-buffered saline
PPARγ: Peroxisome Proliferator-Activated Receptor Gamma
SCFA: short-chain fatty acid
SDS: sodium dodecyl sulfate
SGBS: Simpson-Golabi-Behmel syndrome
TAG: triacylglycerol
TNFα: tumour necrosis factor-alpha
WAT: white adipose tissue
