# Supplementary material for: Effects of selected bioactive food compounds on human white adipocyte function
Source: Nutr Metab (Lond). 2016 Jan 19;13:4. doi: 10.1186/s12986-016-0064-3 (PMC4717570; doi:10.1186/s12986-016-0064-3)
Supplement: Additional file 1: Figure S1. — No effect of BSA and EtOH on glycerol release or IL-6 secretion. Glycerol release (a) and IL-6 secretion (b) after 48 h of treatment with 0.05 % Ethanol (EtOH) and 0.125 μM bovine serum albumin (BSA) relative to untreated cells in conditioned media (n = 2 biological/independent experiments in quintuplicates). Normalized data is adjusted for protein amount and presented as means +/- standard deviation. The absence of statistical significance was determined by Tukey’s test. (PPTX 46 kb) [file 12986_2016_64_MOESM1_ESM.pptx]

## Slide 1
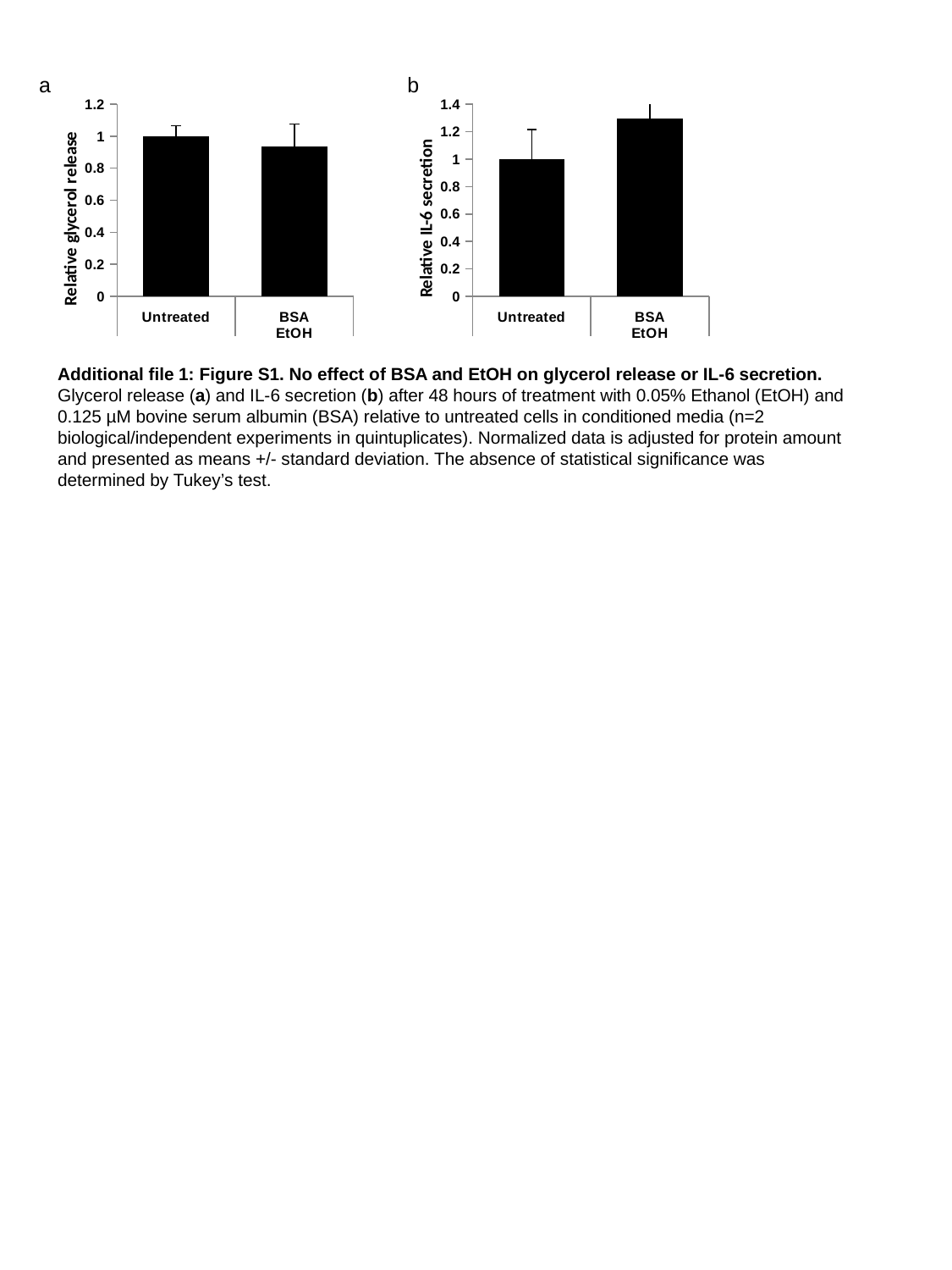

a
b
### Chart
| Category | Glycerol/protein |
|---|---|
| Untreated | 1.0 |
| BSA | 0.935016647494401 |
### Chart
| Category | IL-6/protein |
|---|---|
| Untreated | 1.0 |
| BSA | 1.296274118863838 |Additional file 1: Figure S1. No effect of BSA and EtOH on glycerol release or IL-6 secretion.
Glycerol release (a) and IL-6 secretion (b) after 48 hours of treatment with 0.05% Ethanol (EtOH) and 0.125 µM bovine serum albumin (BSA) relative to untreated cells in conditioned media (n=2 biological/independent experiments in quintuplicates). Normalized data is adjusted for protein amount and presented as means +/- standard deviation. The absence of statistical significance was determined by Tukey’s test.
