# Supplementary material for: Effects of selected bioactive food compounds on human white adipocyte function
Source: Nutr Metab (Lond). 2016 Jan 19;13:4. doi: 10.1186/s12986-016-0064-3 (PMC4717570; doi:10.1186/s12986-016-0064-3)
Supplement: Additional file 2: Figure S2. — Adverse effects of DHA on adipocyte cytotoxicity and IL-6 secretion. Cytotoxicity detection measured as lactate dehydrogenase (LDH) activity in conditioned media after 6 days of treatment with 0.5 μM, 10 μM or 60 μM DHA, relative to control (a). Control, n = 7; 0.5 μM DHA, n = 3; 10 μM and 60 μM DHA, n = 2 biological/independent experiments in at least duplicates. Secretion of IL-6 from human in vitro differentiated adipocytes after 48 h of treatment with 0.5 μM, 5 μM, 10 μM, 20 μM, 30 μM or 60 μM DHA compared to control (b). Control, n = 2 and DHA n = 1 biological/independent experiments in triplicates. Normalized data is adjusted for protein amount and presented as means +/- standard deviation. *p < 0.05, **p < 0.01 and ***p < 0.001 versus control. Statistical significance was obtained by one-way ANOVA with Tukey’s multiple comparisons post hoc test. (PPTX 47 kb) [file 12986_2016_64_MOESM2_ESM.pptx]

## Slide 1
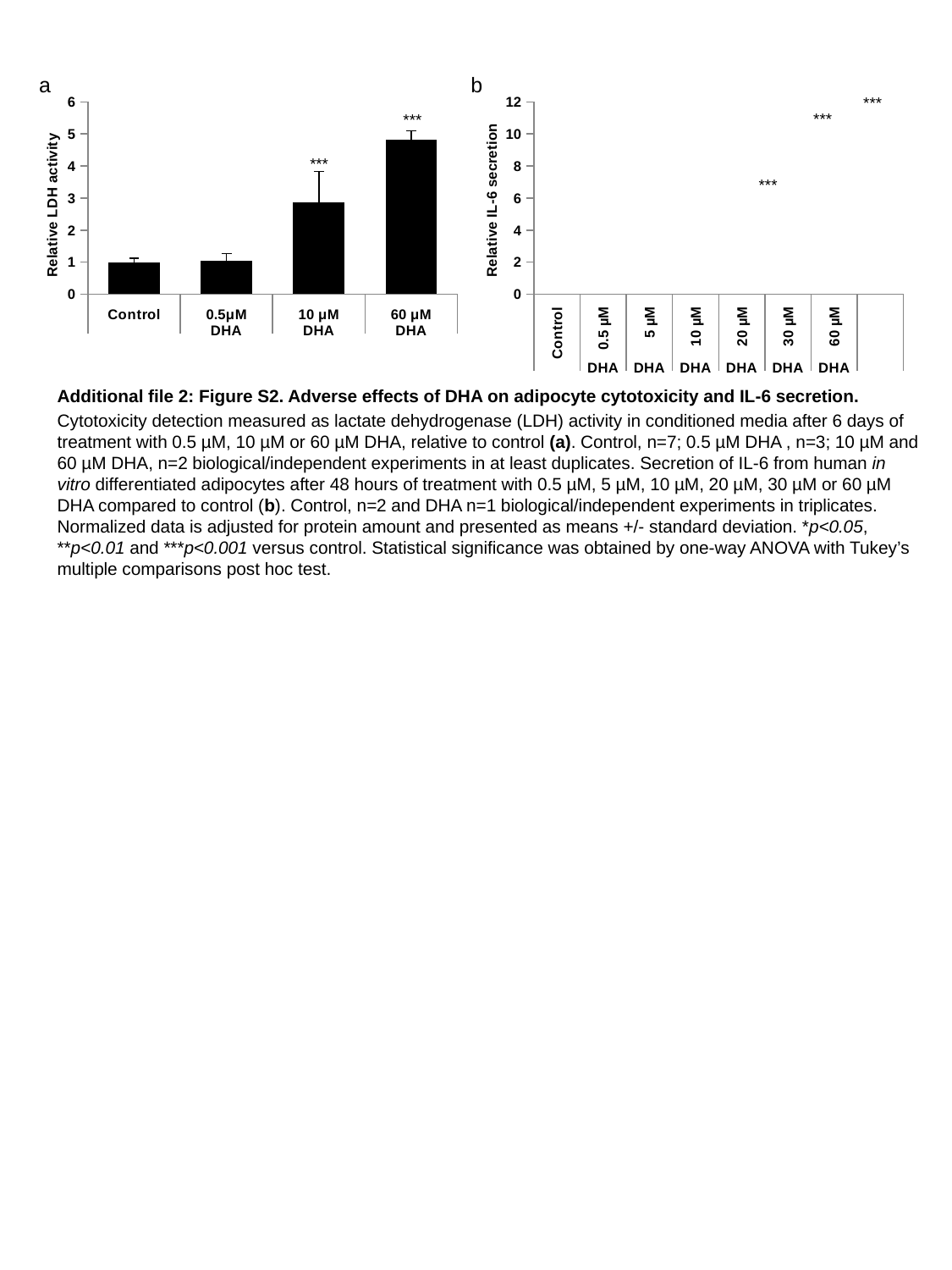

a
b
### Chart
| Category | |
|---|---|
| Control | 0.9999976682222224 |
| 0.5μM | 1.0390841565624582 |
| 10 μM | 2.8528984173720997 |
| 60 μM | 4.821298239749998 |
### Chart
| Category | |
|---|---|
| Control | 1.0 |
| 0.5 µM | 0.8872559301713016 |
| 5 µM | 1.9230904243606102 |
| 10 µM | 3.325084014866642 |
| 20 µM | 7.462185311801492 |
| 30 µM | 11.42381949009015 |
| 60 µM | 15.587500595134182 |***
***
***
***
***
Additional file 2: Figure S2. Adverse effects of DHA on adipocyte cytotoxicity and IL-6 secretion.
Cytotoxicity detection measured as lactate dehydrogenase (LDH) activity in conditioned media after 6 days of treatment with 0.5 µM, 10 µM or 60 µM DHA, relative to control (a). Control, n=7; 0.5 µM DHA , n=3; 10 µM and 60 µM DHA, n=2 biological/independent experiments in at least duplicates. Secretion of IL-6 from human in vitro differentiated adipocytes after 48 hours of treatment with 0.5 µM, 5 µM, 10 µM, 20 µM, 30 µM or 60 µM DHA compared to control (b). Control, n=2 and DHA n=1 biological/independent experiments in triplicates. Normalized data is adjusted for protein amount and presented as means +/- standard deviation. *p<0.05, **p<0.01 and ***p<0.001 versus control. Statistical significance was obtained by one-way ANOVA with Tukey’s multiple comparisons post hoc test.
