# Supplementary material for: Effects of selected bioactive food compounds on human white adipocyte function
Source: Nutr Metab (Lond). 2016 Jan 19;13:4. doi: 10.1186/s12986-016-0064-3 (PMC4717570; doi:10.1186/s12986-016-0064-3)
Supplement: Additional file 3: Figure S3. — Decreased cytotoxicity after treatment with bioactive compounds or metabolites. Lactate dehydrogenase (LDH) activity after 48 h or 6 days treatment with bioactive compounds relative to control in conditioned media. Forty-eight hours; control, n = 9; 0.5 μM DHA, n = 6; AC alone and with DHA, n = 3; 100 μM PI alone and with DHA, n = 4 biological/independent experiments in quadruplicates; 6 days; control, n = 7; 0.5 μM DHA, n = 7; AC alone and with DHA, n = 4; 100 μM PI alone and with DHA, n = 5 biological/independent experiments in duplicates. Normalized data is adjusted for protein amount in each well as a measure of cell amount and presented as means +/- standard deviation. *p < 0.05, **p < 0.01 and ***p < 0.001 versus control. Statistical significance was determined by one-way ANOVA with Tukey’s multiple comparisons post hoc test. (PPTX 44 kb) [file 12986_2016_64_MOESM3_ESM.pptx]

## Slide 1
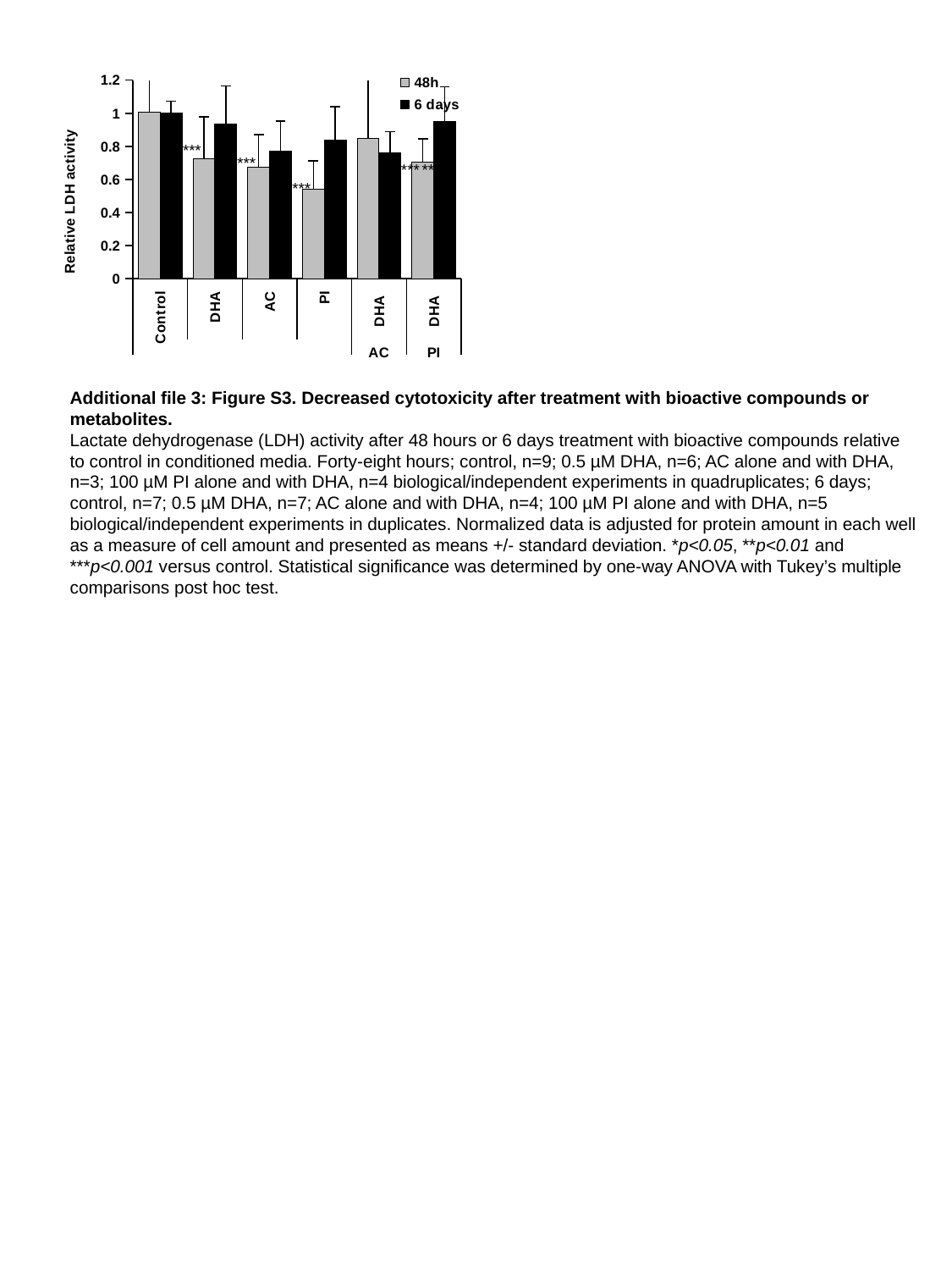

### Chart
| Category | 48h | 6 days |
|---|---|---|
| Control | 1.0079693912956158 | 1.0 |
| DHA | 0.72610717075327 | 0.9328333390570326 |
| AC | 0.6744851894958186 | 0.769936 |
| PI | 0.5421511023238283 | 0.8396203798380526 |
| DHA | 0.8492844023957229 | 0.762233 |
| DHA | 0.7044468841052718 | 0.947942 |***
***
***
***
***
Additional file 3: Figure S3. Decreased cytotoxicity after treatment with bioactive compounds or metabolites.
Lactate dehydrogenase (LDH) activity after 48 hours or 6 days treatment with bioactive compounds relative to control in conditioned media. Forty-eight hours; control, n=9; 0.5 µM DHA, n=6; AC alone and with DHA, n=3; 100 µM PI alone and with DHA, n=4 biological/independent experiments in quadruplicates; 6 days; control, n=7; 0.5 µM DHA, n=7; AC alone and with DHA, n=4; 100 µM PI alone and with DHA, n=5 biological/independent experiments in duplicates. Normalized data is adjusted for protein amount in each well as a measure of cell amount and presented as means +/- standard deviation. *p<0.05, **p<0.01 and ***p<0.001 versus control. Statistical significance was determined by one-way ANOVA with Tukey’s multiple comparisons post hoc test.
